# 塞瑞替尼450 mg随餐服用治疗中国*ALK*阳性非小细胞肺癌患者的安全性及近期疗效分析

**DOI:** 10.3779/j.issn.1009-3419.2020.102.33

**Published:** 2020-08-20

**Authors:** 雨可 田, 甜 田, 萍 余, 莉 任, 友陵 宫, 文秀 姚, 西 张, 俊 殷, 朗 何, 莉 陈, 可 王, 媚娟 黄, 娟 李

**Affiliations:** 1 610041 成都, 电子科技大学医学院附属肿瘤医院, 四川省肿瘤医院肿瘤内科 Department of Medical Oncology, Sichuan Cancer Hospital and Institute, Sichuan Cancer Center, School of Medicine, University of Electronic Science and Technology of China, Chengdu 610041, China; 2 610041 成都, 四川大学华西医院胸部肿瘤科 Department of Thoracic Oncology, West China Hospital, Sichuan University, Chengdu 610041, China; 3 610041 成都, 成都市第三人民医院 Third People's Hospital of Chengdu, Chengdu 610041, China; 4 610041 成都, 四川大学华西医院呼吸与危重症医学科 Department of Respiratory and Critical Care Medicine, West China Hospital, Sichuan University, Chengdu 610041, China; 5 610041 成都, 成都市第五人民医院 Fifth People's Hospital of Chengdu, Chengdu 610041, China; 6 614000 乐山, 乐山市中医院 Hospital of Traditional Medicine of Leshan, Leshan 614000, China

**Keywords:** 塞瑞替尼, 间变性淋巴瘤激酶, 肺肿瘤, 安全性, 疗效, Ceritinib, Anaplastic lymphoma kinase, Lung neoplasms, Safety, Efficacy

## Abstract

**背景与目的:**

间变性淋巴瘤激酶(anaplastic lymphoma kinase, *ALK*)染色体易位为非小细胞肺癌(non-small cell lung cancer, NSCLC)常见的驱动基因。塞瑞替尼为第二代的ALK抑制剂, 可为*ALK*阳性转移性NSCLC患者带来生存获益, 但国内尚无塞瑞替尼用药安全性及疗效的研究报道。因此本研究拟通过真实世界研究来探讨塞瑞替尼450 mg随餐服用治疗中国*ALK*阳性NSCLC患者的安全性及近期疗效。

**方法:**

回顾性分析2018年10月-2019年12月期间就诊于四川省8家医疗机构口服塞瑞替尼450 mg/d随餐治疗的*ALK*阳性NSCLC患者, 收集患者基本信息、治疗期间不良事件(adverse effects, AEs)及疗效数据等资料, 评价其安全性及初步疗效。

**结果:**

研究共纳入109例患者, 随访至2020年1月23日, 中位服药时间为5.87个月(范围：0.4个月-15.7个月), 总体不良事件发生率为89.9%, 3级-4级不良事件发生率为22.9%。最常见AEs(主要为1级-2级)为腹泻(60.6%)、丙氨酸氨基转移酶(alanine aminotransferase, ALT)升高(38.5%)及门冬氨酸氨基转移酶(aspartate aminotransferase, AST)升高(37.6%)。至随访截止, 共45例患者停药, 总体客观缓解率(objective response rate, ORR)为37.6%(95%CI: 28.5%-47.4%), 疾病控制率(disease control rate, DCR)为86.2%(95%CI: 78.3%-92.1%)。

**结论:**

真实世界中, 塞瑞替尼450 mg随餐服用的给药方式在中国*ALK*阳性NSCLC患者中具有良好的安全性及疾病控制率, 但需要更大样本量的前瞻性研究来进一步验证。

## Abstract (en)

**Background and objective:**

Anaplastic lymphoma kinase (*ALK*) rearrangement is a common driver gene of non-small cell lung cancer (NSCLC). Ceritinib is a second-generation ALK inhibitor, which can bring survival benefits to *ALK*-positive metastatic NSCLC. However, few studies focus on the safety and efficacy of ceritinib in China. Therefore, this study intends to investigate the safety and preliminary efficacy of ceritinib 450 mg with meals in Chinese patients with *ALK*-positive NSCLC through a real world study.

**Methods:**

From October 2018 to December 2019, patients with *ALK*-positive NSCLC from 8 medical centers in Sichuan province were recruited in this study. All of these participants received ceritinib 450 mg/d with food. The basic characteristics, adverse effects (AEs) and responses were collected and analyzed in order to evaluate the safety and efficacy of ceritinib.

**Results:**

A total of 109 patients were included in this study. Data cutoff was January 23, 2020. The median duration of treatment exposure was 5.87 mon (range: 0.4 mon-15.7 mon). Total AEs were reported in 98 (89.9%) of 109 patients and grade 3 or 4 AEs were reported in 22.9% of patients. Most common AEs (mainly grade 1 or 2) were diarrhea (60.6%), elevated alanine aminotransferase (ALT)(38.5%) and aspartate aminotransferase (AST)(37.6%). As of data cutoff, 45 patients discontinued ceritinib. The overall response rate (ORR) was 37.6% (95%CI: 28.5%-47.4%) and disease control rate (DCR) was 86.2% (95%CI: 78.3%-92.1%).

**Conclusion:**

The treatment of ceritinib 450 mg with food for Chinese *ALK*-positive NSCLC patients had a good safety profile and favorable DCR in real-world setting. However, this conclusion needs to be further verified by large sample, prospective trials.

肺癌是全球高发的恶性肿瘤, 其中约85%为非小细胞肺癌(non-small cell lung cancer, NSCLC)。因缺乏强有力的早期筛查手段, 约70%-80%患者确诊时已失去手术机会, 接受传统化疗的患者中位生存期不理想^[1]^。随着驱动基因的发现, NSCLC的治疗有了突破性进展。间变性淋巴瘤激酶(anaplastic lymphoma kinase, *ALK*)染色体易位为肺癌的常见驱动基因之一, 在NSCLC中发生率约5%^[2]^。而PROFILE系列研究奠定了第一代ALK酪氨酸激酶抑制剂(tyrosine kinase inhibitor, TKI)克唑替尼在*ALK*阳性进展期NSCLC患者中的治疗地位^[3, 4]^。但患者不可避免的会出现耐药。塞瑞替尼为第二代高选择性的口服ALK-TKI, 体外实验证实其对克唑替尼耐药的肿瘤细胞有明显抑制作用^[5]^。此外, I期/II期临床研究(ASCEND-1及ASCEND-2)初步证实*ALK*阳性NSCLC患者接受塞瑞替尼最大耐受剂量750 mg/d空腹治疗时可获得较好疗效^[6, 7]^。III期临床研究(ASCEND-5及ASCEND-4)亦证实无论在克唑替尼治疗失败后或是一线使用塞瑞替尼, 对比传统化疗, 均可显著延长患者无疾病进展生存时间^[8, 9]^。但既往研究中使用塞瑞替尼750 mg/d空腹口服时患者出现了明显的胃肠道反应, 例如腹泻、恶心及呕吐等, 约60%-80%患者因毒副反应而调整服药剂量、中断或延迟治疗^[8, 9]^, 极大地影响了治疗依从性。为此, ASCEND-8研究对塞瑞替尼的服药方式及剂量进行了探索, 结果^[10, 11]^显示塞瑞替尼450 mg随餐服用与750 mg空腹服用具有相似的药代动力学, 但胃肠道不良反应明显改善, 疗效有进一步获益趋势。然而, 该研究中纳入人群主要为韩裔和高加索人群, 人种的差异所带来的影响仍不得而知。尤其是中国人群的安全性及疗效数据目前尚无相关报道。因此, 本研究回顾性观察了中国人群接受塞瑞替尼450 mg随餐治疗的安全性, 并进行近期疗效的数据分析。

## 1 资料与方法

### 1.1 一般资料

本研究为真实世界观察性、回顾性研究。研究回顾性收集了2018年10月-2019年12月期间就诊于四川省8家医疗机构共109例*ALK*阳性NSCLC患者, 所有患者均接受塞瑞替尼靶向治疗。通过查阅电子病历系统充分采集患者性别、年龄、美国东部肿瘤协作组(Eastern Cooperative Oncology Group, ECOG)评分、吸烟史、病理类型、肿瘤分期、转移部位、基因状态等基本临床特征及既往治疗资料, 通过查询门诊、住院病历记录, 电话及门诊随访收集患者接受塞瑞替尼治疗期间的不良事件(adverse events, AEs)。

### 1.2 纳入及排除标准

纳入标准：①经病理组织学或细胞学确诊为NSCLC; ②美国癌症联合委员会(American Joint Committee on Cancer, AJCC)第八版分期为IIIb期或IV期; ③经逆转录-聚合酶链反应(reverse transcription-polymerase chain reaction, RT-PCR)、Ventana免疫组化法(immunohistochemistry, IHC)、荧光原位杂交法(fluorescence *in situ* hybridization, FISH)或二代测序(next-generation sequencing, NGS)四种方法之一检测确诊*ALK*阳性; ④根据主治医生在临床实践中的判断适合接受塞瑞替尼治疗, 并至少接受1剂塞瑞替尼450 mg随餐口服治疗; ⑤病历资料完整。排除标准：①同时接受塞瑞替尼靶向治疗和其他全身性抗肿瘤治疗; ②无法进行安全性随访的患者。

### 1.3 剂量及用法

所有患者均接受至少1剂塞瑞替尼450 mg随餐口服。所有患者均服用塞瑞替尼至疾病进展、药物不耐受、患者拒绝或死亡而停药。

### 1.4 不良事件及疗效评估

通过查阅电子病历系统、电话及门诊回访等方式对所有患者进行随访。不良事件按照美国国立癌症研究院通用毒性标准(National Cancer Institute Common Terminology Criteria for Adverse Events, NCI-CTCAE)5.0版分级评估, 所有患者均纳入不良事件分析。临床疗效根据实体瘤疗效评价标准(Response Evaluation Criteria in Solid Tumors, RECIST)1.1版评估, 分为完全缓解(complete response, CR)、部分缓解(partial response, PR)、疾病稳定(stable disease, SD)及疾病进展(progressive disease, PD)。客观缓解率(objective response rate, ORR)=(CR+PR)例数/总例数×100.0%, 疾病控制率(disease control rate, DCR)=(CR+PR+SD)/总例数×100.0%。末次随访时间为2020年1月23日。

### 1.5 统计学分析

采用SPSS 25.0软件对数据进行记录分析, 根据数据类型采用相适应的统计量：计量数据为中位数、最小值和最大值, 计数数据为频数和百分比进行描述性统计并计算ORR及DCR的95%置信区间。

## 2 结果

### 2.1 基线情况

研究共纳入109例患者, 基线情况详见表 1。纳入患者的中位年龄52岁(范围：25岁-78岁), 女性患者61例(56.0%), 比例高于男性。大多数患者无吸烟史(86例, 78.9%)。104例患者病理类型为腺癌(95.4%)。107例患者(98.2%)分期为IV期, 其中最常见的远处转移部位为脑(68例, 62.4%)、骨(39例, 35.8%)、肝(18例, 16.5%)及肾上腺(16例, 14.7%)。在基线存在脑转移的患者中, 37例患者既往接受过头部放疗或手术。103例(94.5%)患者为克唑替尼治疗进展或不耐受, 29例(26.6%)进展期患者在接受塞瑞替尼治疗前曾接受过至少1个周期含铂双药化疗。患者从确诊到接受塞瑞替尼治疗的中位时间为16.13个月(范围：0.3个月-90.0个月), 随访至2020年1月23日, 患者中位服药时间为5.87个月(范围：0.4个月-15.7个月)。

**1:** 纳入患者的基线资料(*n*=109) Baseline characteristics of participants (*n*=109)

Characteristics	Data
Age (yr), Median (Range)	52 (25-78)
Gender	
Male	48 (44.0%)
Female	61 (56.0%)
ECOG score	
0-1	66 (60.6%)
> 2	43 (39.4%)
Smoking status	
Ex or current smoker	23 (21.1%)
Never smoked	86 (78.9%)
Pathology	
Adenocarcinoma	104 (95.4%)
Squamous	2 (1.8%)
Others	3 (2.8%)
Stage	
IIIb	2 (1.8%)
IV	107 (98.2%)
Metastatic site	
Brain	68 (62.4%)
Bone	39 (35.8%)
Liver	18 (16.5%)
Chemotherapy in advanced disease	
Yes	29 (26.6%)
No	80 (73.4%)
Local treatment to brain	
Radiotherapy	30 (27.5%)
Surgery	7 (6.4%)
Prior crizotinib	
Yes	103 (94.5%)
No	6 (5.5%)
Others: include adenosquamous carcinoma and non-small cell lung cancer-not otherwise specified (NSCLC NOS); ECOG: Eastern Cooperative Oncology Group.	

### 2.2 安全性

在所有接受塞瑞替尼450 mg随餐服用的109例患者中, 总体AEs发生率为89.9%(98/109)。3级-4级AEs发生率为22.9%(25/109), 其中最常见的3级-4级AEs为谷氨酰转肽酶(gamma-glutamyltransferase, GGT)升高(11.0%)、丙氨酸氨基转移酶(alanine aminotransferase, ALT)或门冬氨酸氨基转移酶(aspartate aminotransferase, AST)升高(5.5%)。

常见不良事件如表 2所示。所有不良事件中发生率最高的为腹泻(60.6%), 多数患者经口服止泻药物和肠道益生菌后症状可缓解。2例患者出现3级腹泻, 其中1例患者因腹泻减量至300 mg/d, 1例患者选择永久停药。其他消化道不良反应包括食欲下降(31.2%)、呕吐(24.8%)、恶心(23.9%)及上腹痛(15.6%), 主要实验室检查异常为肝功能异常[包括ALT升高(38.5%)、AST升高(37.6%)、GGT升高(29.4%)]、血肌酐升高(20.2%)及贫血(15.6%)。其他常见AEs还包括皮肤毒性(22.0%)、乏力(19.3%)及体重下降(11.0%)。本研究中还观察到其他少见不良事件, 如表 3所示。研究中观察到较低的心脏毒性及视觉异常发生率, 无新发间质性肺炎病例。绝大部分AEs(包括3级-4级)均可通过对症支持治疗进行管理, 患者耐受性好。10例患者(9.2%)因3级-4级AEs而中断、减量或永久停药。无治疗相关死亡病例。

**2:** 常见不良事件汇总(不计药物相关性, 所有级别 > 10%或3级-4级 > 2%) Summary of common adverse events (regardless of drug relationship, > 10% of patients at all grades or > 2% of patients at grade 3/4)

Adverse event	All grades	Grade 1/2	Grade 3/4
Any AE	98 (89.9%)	73 (67.0%)	25 (22.9%)
Diarrhea	66 (60.6%)	64 (58.7%)	2 (1.8%)
ALT increased	42 (38.5%)	36 (33.0%)	6 (5.5%)
AST increased	41 (37.6%)	35 (32.1%)	6 (5.5%)
Decreased appetite	34 (31.2%)	31 (28.4%)	3 (2.8%)
GGT increased	32 (29.4%)	20 (18.3%)	12 (11.0%)
Vomiting	27 (24.8%)	24 (22.0%)	3 (2.8%)
Nausea	26 (23.9%)	26 (23.9%)	0 (0.0%)
Skin toxicity	24 (22.0%)	24 (22.0%)	0 (0.0%)
Blood creatinine increased	22 (20.2%)	21 (19.3%)	1 (0.9%)
Fatigue	21 (19.3%)	21 (19.3%)	0 (0.0%)
Upper abdominal pain	17 (15.6%)	15 (13.8%)	2 (1.8%)
Anemia	17 (15.6%)	17 (15.6%)	0 (0.0%)
Weight loss	12 (11.0%)	11 (10.1%)	1 (0.9%)
ALT: alanine aminotransferase; AST: aspartate aminotransferase; GGT: gamma-glutamyl transferase; skin toxicity: include rash, dry skin or skin itch.			

**3:** 罕见不良事件汇总(不计药物相关性, 发生率 < 10%) Summary of rare adverse events (regardless of drug relationship, < 10% of patients)

Adverse event	All grades	Grade 1/2	Grade 3/4
Non-cardiac chest pain	10 (9.2%)	9 (8.3%)	1 (0.9%)
Creatine kinase increased	7 (6.4%)	7 (6.4%)	0 (0.0%)
Cough	6 (5.5%)	6 (5.5%)	0 (0.0%)
Back pain	6 (5.5%)	6 (5.5%)	0 (0.0%)
Thrombocytopenia	6 (5.5%)	6 (5.5%)	0 (0.0%)
Dyspnea	5 (4.6%)	4 (3.7%)	1 (0.9%)
Neutropenia	4 (3.7%)	4 (3.7%)	0 (0.0%)
Leukopenia	4 (3.7%)	4 (3.7%)	0 (0.0%)
Peripheral edema	3 (2.8%)	3 (2.8%)	0 (0.0%)
Hyperglycemia	2 (1.8%)	0 (0.0%)	2 (1.8%)
Headache	2 (1.8%)	2 (1.8%)	0 (0.0%)
Dizzy	2 (1.8%)	2 (1.8%)	0 (0.0%)
Tinnitus	2 (1.8%)	2 (1.8%)	0 (0.0%)
Stomatitis	2 (1.8%)	2 (1.8%)	0 (0.0%)
Proteinuria	2 (1.8%)	2 (1.8%)	0 (0.0%)
Limb numbness	2 (1.8%)	2 (1.8%)	0 (0.0%)
Visual disturbance	2 (1.8%)	2 (1.8%)	0 (0.0%)
Prolonged QT	2 (1.8%)	2 (1.8%)	0 (0.0%)
Bradycardia	1 (0.9%)	1 (0.9%)	0 (0.0%)

### 2.3 剂量调整情况

服药期间, 共有8例患者(7.3%)因AEs而下调塞瑞替尼治疗剂量至300 mg/d, 具体原因如下：6例因消化道不良反应(如腹泻、腹痛、恶心、呕吐或食欲下降)而调整剂量; 1例因肾功能损伤(血肌酐升高3级及蛋白尿2级)而调整剂量; 1例因出现2级呼吸困难而调整剂量。

服药期间, 共有5例患者(4.6%)因ALT、AST或血肌酐升高等AEs而中断药物治疗, 经积极对症处理好转后恢复用药。3例患者(2.8%)因AEs而自行永久停药, 主要原因为消化道不良反应(2例)及乏力(1例)。具体见表 4。

**4:** 患者服药期间的用药剂量调整情况 Dosage adjustment of patients during medication

Index	Ceritinib (*n*=109)
Median duration of treatment exposure	5.87 mon
Dose adjustment or interruption	12 (11.0%)
AE requiring treatment discontinuation	3 (2.8%)
AE requiring dose adjustment	8 (7.3%)
AE requiring dose interruption	5 (4.6%)
GI toxicity requiring dose adjustment or interruption	6(5.5%)
Vomiting	3(2.8%)
Nausea	2(1.8%)
Decreased appetite	2(1.8%)
Upper abdominal pain	2(1.8%)
Diarrhea	1(0.9%)
GI toxicity requiring treatment discountinuation	2(1.8%)
AE: adverse event; GI: gastrointestinal.	

### 2.4 近期疗效

至随访截止, 45例患者已停药, 主要停药原因为疾病进展(42例, 38.5%)。109例患者中, 103例患者完成至少一次肿瘤评估, 6例患者在服药后首次疗效评价前死亡, 未收集到疗效评价信息。无患者达到CR, 41例患者疗效评价PR, 53例患者SD。初步疗效数据显示, 所有患者总体ORR为37.6%(95%CI: 28.5%-47.4%), DCR为86.2%(95%CI: 78.3%-92.1%)。

## 3 讨论

塞瑞替尼在2014年首次被美国食品药品监督管理局(Food and Drug Administration, FDA)获批用于经克唑替尼治疗进展或不耐受的进展期*ALK*阳性NSCLC患者, 其推荐剂量为750 mg空腹口服。但在这一剂量水平下3级-4级不良事件的发生率高达70%-80%, 胃肠道毒性发生率更是不容小觑(腹泻：72%-86%, 恶心：66%-83%, 呕吐：52%-67%), 因此极大地限制了药物的临床应用^[12]^。如何在保证药物疗效不受影响的同时降低药物胃肠道不良反应发生率已成为重要的临床问题。

既往研究^[13-16]^曾报道过酪氨酸激酶抑制剂和食物同服可减少药物的胃肠道不良反应并且增加药物系统性暴露, 塞瑞替尼也曾在健康受试者中进行了类似研究, 结果一致。基于此, Cho等^[10, 11]^比较了在韩裔及高加索人群中, 初治和经治的*ALK*阳性NSCLC患者随机接受塞瑞替尼450 mg随餐、600 mg随餐和750 mg空腹三种不同的给药方法之间, 药物疗效和不良反应发生率的差异。结果显示三组患者中塞瑞替尼一线治疗的ORR分别为78.1%、72.5%和75.7%, 组间差异无统计学意义, 但在药物安全性方面, 三组患者药物减量的比例分别为24.1%、65.1%和60.9%, 胃肠道不良反应的发生率分别为75.9%、82.6%和91.8%。因此, Cho等^[10, 11]^研究结果显示450 mg随餐剂量组具有最低的药物剂量调整率及胃肠道不良反应发生率。随后2018年塞瑞替尼在我国获批的适应证剂量也为450 mg随餐服用, 每日1次。目前, 塞瑞替尼在国内上市时间已经1年余, 但尚未有塞瑞替尼治疗相关的安全性数据及疗效数据报道。本研究在国内首次探讨了塞瑞替尼450 mg随餐口服治疗中国人群中*ALK*阳性NSCLC的不良反应和初步疗效数据。

本研究结果显示服用塞瑞替尼后最常见的AEs为腹泻(60.6%)、ALT或AST升高(约38.0%)、食欲下降(31.2%)、GGT升高(29.4%)、呕吐(24.8%)和恶心(23.9%)。常见不良反应谱与既往以高加索人群为主的ASCEND系列研究类似。ASCEND-2研究^[7]^纳入了140例经克唑替尼及化疗后进展的*ALK*阳性NSCLC, 其中亚洲人群约占40%, 所有患者均接受塞瑞替尼750 mg/d空腹口服。与ASCEND-2研究相比, 本研究中总体AEs发生率降低(89.9% *vs* 100.0%), 3级-4级AEs发生率明显降低(22.9% *vs* 71.4%)。改变给药模式后下降最明显的是消化道不良反应(呕吐：24.8% *vs* 62.9%, 恶心：23.9% *vs* 81.4%, 腹泻：60.6% *vs* 80.0%), 3级-4级消化道毒性下降的尤为明显(呕吐：2.8% *vs* 4.3%, 恶心：0.0% *vs* 6.4%, 腹泻：1.8% *vs* 6.4%)。因此, 同塞瑞替尼750 mg空腹服用相比, 450 mg随餐的给药模式可明显降低中国人群中塞瑞替尼导致的消化道不良反应。

在药物依从性方面, 本研究中观察到的因AEs而导致药物剂量调整或中断的发生率为11%, 主要原因为胃肠道毒性(腹泻、腹痛、恶心、呕吐或食欲下降), 发生率为5.5%。而在ASCEND-2研究^[7]^中, AEs导致的药物暂停或剂量调整的比例超过50%, 主要原因也是消化道不良反应, AEs导致停药的发生率达到7.9%, 显著高于本研究(2.8%)。在同样为450 mg随餐口服的ASCEND-8研究^[11]^中, AEs导致药物剂量调整、暂停和停药的发生率分别为17.6%、45.4%及7.4%, 也明显高于本研究。然而, 对停药原因的分析显示, 本研究中2例患者因出现胃肠道毒性而主观意愿选择永久停药。因此, 研究观察到因胃肠道毒性而停药的发生率略高于ASCEND-8研究(1.8% *vs* 0.0%), 考虑可能因为临床试验中具有严格的停药标准, 而真实世界中患者的停药受多种因素影响(如本研究中受患者主观意愿影响), 两者的差异最终导致了停药原因的不同。总体而言, 本研究观察到在中国人群中塞瑞替尼450 mg随餐服用的给药方案可显著提高患者的药物依从性。

在人种差异方面, 本研究同以韩国人群和高加索人群为研究对象的ASCEND-8研究相比较, 同样是塞瑞替尼450 mg随餐服用的给药模式, 不管是在AEs的总体发生率(89.9% *vs* 99.1%)或3级-4级AEs的发生率(22.9% *vs* 64.8%)方面, 中国人群的安全性数据都更为优异^[11]^。而消化道不良反应的发生率方面, 腹泻的发生率与ASCEND-8研究类似(60.6% *vs* 57.4%), 而呕吐(24.8% *vs* 38.9%)及恶心(23.9% *vs* 41.7%)发生率均较ASCEND-8研究降低。食欲下降及皮肤毒性似乎略高于ASCEND-8研究中所报道数据, 提示中国人群可能具有和其他人群不完全一致的药物毒性谱, 需要在后续的研究及临床实践中进行进一步的探索。

本研究也观察了中国人群中塞瑞替尼450 mg随餐用于*ALK*阳性NSCLC的初步疗效数据。至随访截止, 总体ORR为37.6%(95%CI: 28.5%-47.4%), DCR为86.2%(95%CI: 78.3%-92.1%)。同ASCEND-2研究相比较, 初步的疗效数据相近(ORR: 37.6% *vs* 38.6%; DCR: 86.2% *vs* 77.1%)^[7]^。而与ASCEND-5日本人群亚组分析^[17]^相比, 本研究亦获得相类似的DCR(86.2% *vs* 90.9%)。由于本研究样本量较小, 随访时间较短, 进一步的疗效数据需要在后续随访中继续收集。

总体而言, 塞瑞替尼450 mg随餐服用的给药方式在中国人群中是安全、有效的。同既往研究中750 mg空腹服用的给药方法相比, 塞瑞替尼450 mg随餐服用能够显著降低患者总体不良事件的发生率, 特别是消化道不良反应的发生, 同时还提高了患者的药物依从性。初步的疗效数据显示, 塞瑞替尼450 mg随餐服用治疗*ALK*阳性NSCLC具有较好的DCR, 但还需要更大样本量的前瞻性研究来进一步验证和证实。
